# Eco-evolutionary dynamics of a shifting porcine parvoviruses (PPV1–PPV8) ecosystem reveal dichotomous selection pressures

**DOI:** 10.1186/s13567-026-01756-4

**Published:** 2026-04-18

**Authors:** Changchun Zhang, Zhiying Xu, Guangyu Liu, Huixin Li, Yunlan Ling, Lele Zhao, Xiaolong Xu, Yimin He, Ning Ding, Guihong Zhang, Yankuo Sun

**Affiliations:** 1 Guangdong Provincial Key Laboratory of Zoonosis Prevention and Control, College of Veterinary Medicine, South China Agricultural University, Guangzhou, China; 2 Maoming Branch, Guangdong Laboratory for Lingnan Modern Agriculture, Guangdong, China; 3 State Key Laboratory of Vaccines for Infectious Diseases, Xiang An Biomedicine Laboratory, State Key Laboratory of Molecular Vaccinology and Molecular Diagnostics, National Innovation Platform for Industry-Education Integration in Vaccine Research, School of Public Health, Xiamen University, Xiamen, China; 4 Nuffield Department of Medicine, Pandemic Sciences Institute, University of Oxford, Oxford, UK; 5 Hipra Biotech Consulting (Beijing) Co., Ltd., Beijing, China; 6 Guangzhou Customs Technology Center, Guangzhou, 510623 China; 7 Li Ka Shing Centre for Health Information and Discovery, Big Data Institute, University of Oxford, Oxford, UK

**Keywords:** Porcine parvoviruses, viral ecosystems, eco-evolutionary dynamics, co-infection, vaccine-induced selection

## Abstract

**Abstract:**

Porcine parvoviruses (PPVs) constitute a complex viral ecosystem. However, the genetic structure and evolutionary dynamics of currently circulating PPV populations remain poorly characterized. A large-scale genomic survey in China (1,055 genomes; 863 samples; 2021–2024) reveal a profound ecosystem shift, with PPVs detected in 212/863 samples (24.57%) Novel species (PPV7, 16.45%; PPV6, 9.62%; and PPV2, 8.82%) now dominate the niche previously occupied by classical PPV1 (3.13%). This new landscape is defined by pervasive multi-species co-infection (129/212, 60.85%), including 61 cases with ≥ 3 species and 7 pigs co-infected with ≥ 5 species, characterized by non-random associations, where even low-prevalence species such as PPV1 and PPV5 exhibit a high propensity for co-infection (permutation test: PPV1 *p* < 0.001; PPV5 *p* = 0.002). This high-transmission environment provides ample opportunities for frequent intra-species recombination, which powerfully accelerates variation within the primary antigenic region (ORF2). This study uncovered dichotomous evolutionary dynamics driven by contrasting immune pressure. The dominant naturally circulating species were constrained by strong purifying selection. In stark contrast, vaccine-targeted PPV1 followed a distinct path driven by strong positive selection of key surface epitopes (e.g., VP2 site 435), suggesting an adaptation to evade vaccine-induced immunity. Collectively, our work deconstructs how natural selection and vaccine filtering impose distinct selective regimes on the viral population, establishing a new framework for evolutionarily aware surveillance and rational design of next-generation vaccines.

**Graphical abstract:**

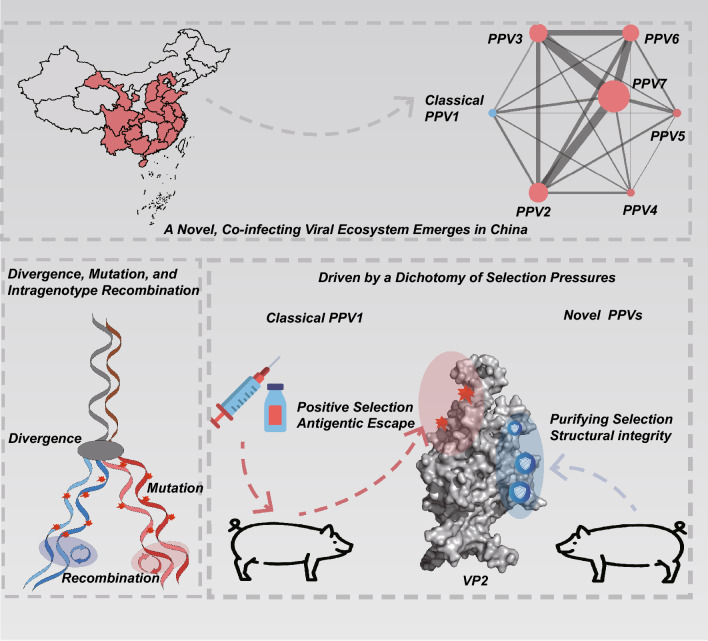

**Supplementary Information:**

The online version contains supplementary material available at 10.1186/s13567-026-01756-4.

## Introduction

The *Parvoviridae* family comprises a diverse group of small, non-enveloped, single-stranded DNA viruses that infect a wide range of animal species, often resulting in host-dependent pathogenic outcomes [1, 2]. Among these, porcine parvoviruses (PPVs), a diverse group of co-circulating species (PPV1–PPV8), represent important etiological agents of reproductive failure in the global swine industry, with PPV1 being definitively established as a causative pathogen, whereas the pathogenicity of PPV2–PPV8 remains under investigation. The resulting syndrome, known as SMEDI (Stillbirth, Mummification, Embryonic Death, and Infertility), primarily caused by PPV1, causes significant economic losses to the swine industry worldwide. By drastically reducing litter size and compromising herd fertility, PPV1 acts as a persistent barrier to achieving optimal production targets with cumulative global losses [3–6].

Since the initial identification of classical PPV1, our understanding of PPV diversity has expanded dramatically with the discovery of seven additional viral species (PPV2–PPV8) over the past two decades [7–14]. Under the current International Committee on Taxonomy of Viruses (ICTV) classification, this highly divergent group of pathogens does not represent genetic variants of a single virus, but rather comprises distinct viral species spanning multiple genera within the *Parvovirinae* (e.g., *Protoparvovirus*, *Tetraparvovirus*, and *Copiparvovirus*) and *Hamaparvovirinae* (*Chapparvovirus*) subfamilies (Table 1) [14–17]. Recognizing this extensive taxonomic diversity is essential, as it has led to the formation of a complex viral ecosystem within swine populations, in which co-infection with multiple distinct PPV species is now a common ecological feature [13].

**Table 1:** **Taxonomic classification of PPVs based on current ICTV guidelines**

Common name	Subfamily	Genus	Species name (ICTV/proposed)
PPV1	*Parvovirinae*	*Protoparvovirus*	*Ungulate protoparvovirus 1*
PPV2	*Parvovirinae*	*Tetraparvovirus*	*Ungulate tetraparvovirus 3*
PPV3	*Parvovirinae*	*Tetraparvovirus*	*Ungulate tetraparvovirus 2*
PPV4	*Parvovirinae*	*Copiparvovirus*	*Ungulate copiparvovirus 2*
PPV5	*Parvovirinae*	*Copiparvovirus*	*Ungulate copiparvovirus 1 (provisional)*
PPV6	*Parvovirinae*	*Copiparvovirus*	*Ungulate copiparvovirus 4*
PPV7	*Hamaparvovirinae*	*Chapparvovirus*	*Ungulate chapparvovirus 1*
PPV8	*Parvovirinae*	*Protoparvovirus*	Unassigned / Proposed*

Despite extensive global research, significant knowledge gaps persist regarding the contemporary epidemiology, species distribution, and co-infection dynamics of PPVs circulating in China, which is one of the world's largest swine producers [18]. While previous studies have noted divergence between Chinese strains and global references, and even demonstrated significant intra-species evolution [19, 20], these efforts were often limited by small sample sizes or the constraints of traditional PCR or Sanger sequencing methods, which are ill-suited for characterizing complex mixed infections and resolving fine-scale evolutionary patterns [21].

The challenge of controlling this viral ecosystem is compounded by a fundamental mismatch between the evolutionary strategy of the pathogen and current intervention methods. As an ssDNA virus lacking proofreading, PPV possesses immense evolutionary potential, driven by high mutation rates and frequent recombination [22, 23]. The primary target for this evolution is the capsid (VP) protein region, as it is an immunodominant target for host antibody responses and is critical for host–cell interactions [24–27]. Historically, control strategies have focused on a single-species approach using vaccines based on the classical PPV1 strain. However, the long-term efficacy of this strategy has been questioned, as evidenced by the increasing prevalence of PPV1 among vaccinated herds in Europe [28]. This highlights a critical paradox: current control strategies are largely directed at a single historical species, whereas the pathogen itself operates as a rapidly evolving multi-species collective. Therefore, it is crucial to understand how the viral community evolves in response to the powerful and uniform selective pressures imposed by vaccination.

To resolve this, we implemented a large-scale genomic surveillance approach to deconstruct the eco-evolutionary dynamics of diverse porcine parvoviruses (PPV1–PPV8) in China. Specifically, we aimed not only to delineate contemporary epidemiological and genomic features, but, more importantly, to establish a comparative evolutionary framework. By contrasting the evolutionary trajectories of vaccine-targeted PPV1 against dominant, naturally evolving species that served as internal controls, we sought to untangle the contrasting evolutionary patterns of natural selection versus vaccine-induced pressure. This approach allowed us to test fundamental hypotheses regarding how a complex viral community responds to targeted, artificial interventions, providing a clearer path toward evolution-informed control strategies.

## Materials and methods

### Ethics statement and sample collection

Between January 2021 and June 2024, 863 serum samples were collected from breeding pigs (replacement gilts and multiparous sows) on intensive commercial farms across 18 provincial-level regions in China, including Anhui, Fujian, Gansu, Guangdong, Guangxi, Guizhou, Hainan, Hebei, Henan, Hubei, Jiangsu, Jiangxi, Shandong, Shanxi, Sichuan, Zhejiang, Chongqing, and Yunnan. Samples were obtained through routine epidemiological surveillance submissions. Based on accompanying submission records, most animals had no obvious acute clinical signs at the time of sampling. Vaccination information was extracted from submission forms and farm records when provided; herd-level records indicated routine use of commercial inactivated vaccines, but individual vaccination dates and vaccination-to-sampling intervals were not consistently available in this cross-sectional surveillance. All sampling procedures were approved by the Animal Ethics Committee of South China Agricultural University (SCAU-AEC-2022A010) and were conducted in accordance with relevant guidelines.

### Library preparation and genome sequencing

Viral nucleic acids were extracted from these samples using the VAMNE Magnetic Pathogen DNA/RNA Kit (RM602-01; Vazyme, China). Libraries were prepared using the VAHTS DNA & RNA Library Prep Kit for MGI (NCM601-01; Vazyme, China) following the manufacturer’s instructions. The quality and concentration of the libraries were assessed using a Nanodrop Lite spectrophotometer (ND-LITE-PR; Thermo Fisher Scientific, USA) and a Qubit 4.0 Fluorometer (Q33238; Invitrogen, USA). Libraries were then processed and sequenced on the MGI platform (BGI; Shenzhen, China) to generate NGS data.

### Data processing and statistical analysis

Raw sequencing reads were processed using Trimmomatic (v0.39) to remove adapter sequences and filter low-quality reads (quality score < 30) [29]. The filtered reads were assembled into contigs using MEGAHIT (v1.2.9) [30]. To screen for PPVs within this metagenomic dataset, the assembled contigs were analyzed using BLAST, DIAMOND, and Kraken2 [31–33]. Samples containing identified PPV contigs were determined to be positive, and these viral sequences were subsequently extracted for further analysis. Whole-genome sequences were constructed based on the PPV contigs. Primers targeting specific regions were designed, and the corresponding amplicons were processed via Sanger sequencing to verify the sequences and correct any gaps or variant sites from sequencing errors.

The prevalence of each PPV species was calculated with 95% CI determined using the Clopper–Pearson exact method [34]. Co-infection patterns were analyzed by constructing a weighted co-occurrence network, in which each species was represented as a node. An edge was drawn between any two species *i* and *j* if they co-occurred in at least one host, and the edge weight (*W*_*ij*_) was defined as the total count of co-infected hosts (*C*_*i,j*_). To quantify the intrinsic co-infection propensity of each species while controlling for its prevalence, normalized node strength was calculated. This metric provides a clearer view of the co-infection propensity by representing the average number of pairwise co-infection events per host for a given species *i*, calculated as follows [35].

$$Normalized Strength=\frac{{\sum }_{j\ne i}{C}_{i,j}}{{N}_{I}}$$

where *N*_*i*_ is the total number of hosts infected with species *i*. The statistical significance of the normalized strength values was assessed using a permutation test (10 000 iterations), in which the empirical *p*-values were derived from a null distribution generated by randomly permuting species labels across the sample-by-species matrix. Network visualization was performed using Cytoscape (v3.9.1) [36].

### Sequence alignment and phylogeny reconstruction

The CDSs for each PPV species were downloaded from GenBank as references, whereas the sequences obtained in this study were treated as datasets (Additional file 3). Multiple sequence alignments for each species were performed using MAFFT (v7.525) [37]. To ensure accurate phylogenetic reconstruction, all ambiguously aligned regions were removed using trimAl (v1.4) [38]. Similarities among CDSs, both inter- and intra-species, were assessed using BioAider (v7.525) [39]. To further investigate the evolutionary history of each PPV species, phylogenetic relationships were examined across alignments for each of the seven major species and one global alignment including all species. Phylogenetic trees were constructed using the maximum likelihood (ML) approach in the IQ-TREE (v2.2.0) software, applying the best-fit model for each dataset and 1000 bootstrap replicates. The results were visualized using iTOL (v7) [40, 41].

Additionally, to investigate the antigenic divergence of PPV1, a separate high-resolution phylogenetic analysis was performed based on VP2. VP2 coding sequences from all newly obtained PPV1 genomes (*n* = 17) and three reference inactivated PPV1 vaccine strains (WH-1, S-1, and CP-99) were extracted and aligned using MAFFT. An ML tree was subsequently constructed using the same methodology as for the IQ-TREE (best-fit model, 1000 bootstraps). This VP2-specific tree was visualized and annotated along with a heatmap of the key amino acid substitutions using the ggtree package in R (v4.3.1) [42].

### Inter-species and intra-species recombination analysis

Recombinant events, including both inter- and intra-species recombination, were identified using the Recombination Detection Program 4 (RDP4) (v4.101), employing methods such as RDP, Chimaera, BootScan, 3Seq, GENECONV, MaxChi, and SiScan [43]. Each recombinant event was considered reliable only if it was supported by at least three detection methods and all associated *p*-values were < 0.001 [44]. All breakpoints were further validated through recombination analysis of the major and minor parental strains using SimPlot [45].

### Positional conservation and selection pressure analysis

Two sets of analyses were performed to quantify the evolutionary pressure and sequence variability acting on the PPV capsid. To identify high-frequency mutation sites (hotspots) for each species, nucleotide sequence datasets (outlined in Section "Sequence alignment and phylogeny reconstruction") were translated into amino acid sequences using MEGA X (v10.2.6) [46]. Shannon entropy was then calculated at both the nucleotide and amino acid levels for each alignment position using the following formula [47].

$$H_{s} \left( {\mathrm{p}} \right) = - \mathop \sum \nolimits_{i = 1}^{H} p_{i} \log_{2} (p_{i} )$$

where, *pi* represents the frequency of each characteristic (nucleotide or amino acid) at a given site. The resulting entropy values, which increased with residue diversity, were visualized using ggplot2 (v3.5.2) [48]. Mutation hotspots were defined quantitatively to highlight the most hypervariable sites for visualization. Second, to detect sites under positive selection, specifically in vaccine-type PPV1, VP2 coding sequences from all newly obtained PPV1 genomes (*n* = 17) and three reference inactivated PPV1 vaccine strains (WH-1, S-1, and CP-99) were codon-aligned. This alignment was then analyzed using the FUBAR (posterior probability > 0.9) and FEL (*p* < 0.1) methods on the Datamonkey web server, with thresholds set to standard values recommended for exploratory analysis to identify potential candidate sites for selection [49].

### Structural modeling and feature mapping

To visualize key evolutionary features in a three-dimensional context, prototypic or well-characterized reference strains for each major species were selected for structural reconstruction using AlphaFold2 (v2.3.2) [50]. These included PPV1 NADL-2 (GenBank: NC001718.1), PPV2 Brazil (GenBank: KY586144.1), PPV3 Brazil (GenBank: KY586145.1), PPV4 (GenBank: GQ387499), PPV5 MI216 (GenBank: JX896318.1), PPV6 Br (GenBank: KY094494.1), and PPV7-GX23-1998 (GenBank: MN326273.1). Potential linear B-cell epitopes on these structures were predicted using BepiPred-2.0 server [51]. All structural visualizations and site mappings were performed using PyMOL (v4.6) [52]. This framework was utilized for two primary visualization tasks: (1) for pan-species analysis, mutation hotspots identified by Shannon entropy were mapped onto each of the seven representative structures to assess their location relative to predicted epitopes; and (2) for in-depth PPV1 analysis, the identified positively selected sites, along with dominant amino acid substitutions, were mapped onto the WH-1 reference structure, and sequence logos of epitope regions were generated using WebLogo (v3.7.9) [53].

## Results

### High prevalence and a shifting species landscape of PPVs in China

**Figure 1:**
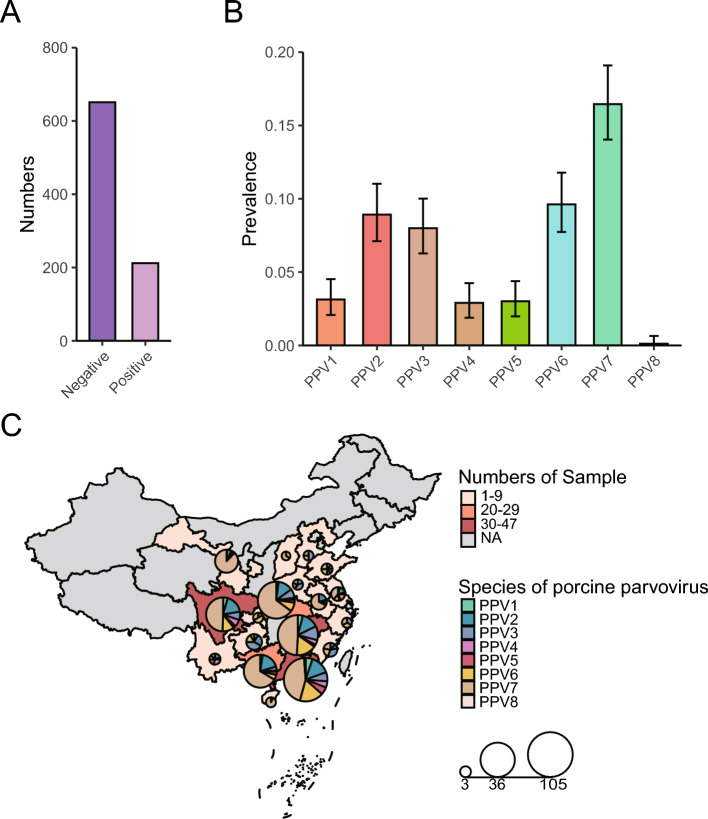
**Epidemiology and geographic distribution of porcine parvoviruses (PPVs) species**. A Numbers of PPVs positive samples and PPVs negative samples. B Prevalence of each PPV species among all sequenced samples. C Geographic distribution of the PPVs positive specimens. The color of the province on the map represents the number of samples in the province. The color from light to dark indicates the increase in the number of samples, and the color gray indicates that no sample was collected in the province. The number of PPVs of each species in each province was counted and plotted as a pie chart. The size of the circle represents the number of sequences that are recognized as PPV-derived, and each color in the circle represents a single species

Of the 863 swine serum samples collected from 18 Chinese provinces between January 2021 and June 2024, 212 tested positive for PPVs, yielding an overall prevalence of 24.57% (95% CI: 21.7%–27.6%) (Figure 1A). Metagenomic analysis of these samples revealed the circulation of all eight known PPV species (PPV1–PPV8). The contemporary species landscape was dominated by PPV7 (found in 16.45% of all samples; 95% confidence interval [CI]: 14.0%–19.1%), PPV6 (9.62%; 95% CI: 7.7%–11.8%), and PPV2 (8.82%; 95% CI: 7.0%–10.9%), with PPV3 also being highly prevalent (7.99%; 95% CI: 6.3%–10.0%) (Figure 1B). In stark contrast, the classic vaccine-type PPV1 (3.13%; 95% CI: 2.1%–4.5%), along with PPV4 (2.90%; 95% CI: 1.9%–4.2%) and PPV5 (3.01%; 95% CI: 2.0%–4.4%), were detected at substantially lower frequencies. Notably, we identified PPV8 in a single sample (prevalence, 0.47%; 95% CI: 0.0%–0.6%).

Geographically, the PPVs were widespread across the major swine-producing regions surveyed (Figure 1C). A key finding was the frequent cocirculation of multiple species within the same province. For instance, regions such as Guangdong, Guangxi, and Sichuan exhibit high viral diversity, and PPV7 is typically the most abundant species, often found alongside PPV6 and PPV2. This extensive co-circulation of diverse species across China underscores the complex viral ecosystem and sets the stage for the high frequency of co-infections observed in the present study.

### Analysis of co-infection patterns reveals non-random species associations

**Figure 2:**
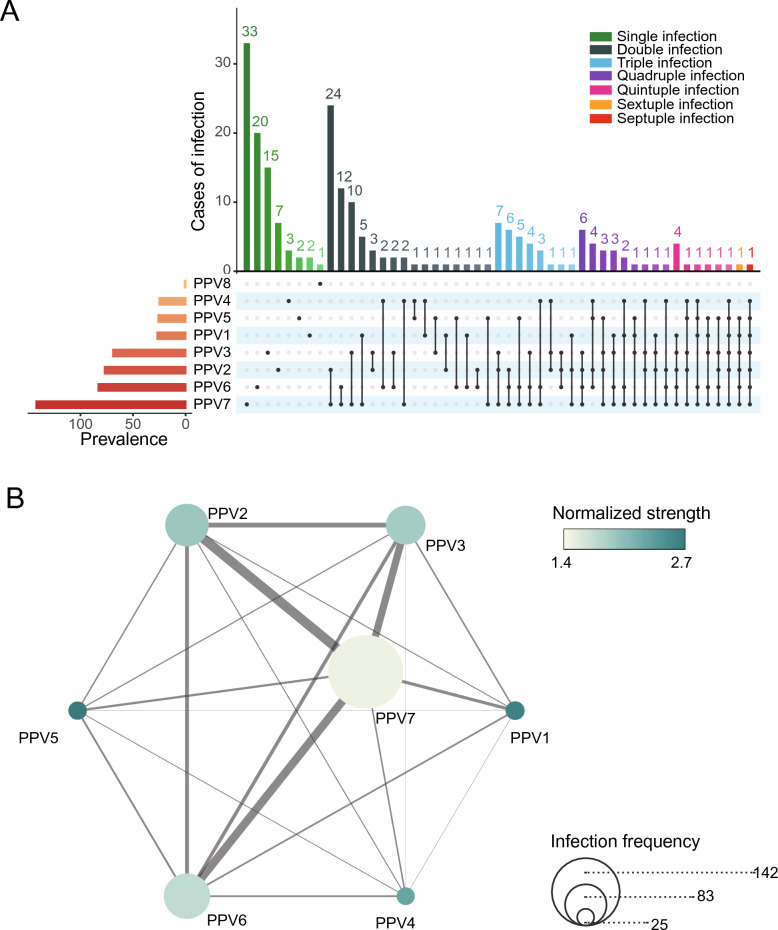
**Patterns and network analysis of PPVs co-infections**. A Distribution of co-infections with different PPVs specie s within positive samples and co-infection frequencies among different species. Different species and co-infection statuses are shown in separate colors. B Correlation between co-infection occurrence among different PPVs species. The color gradient in teal color gradient represents increasing normalized strength, with the value in each cell indicating the correlation coefficient. The thickness of the connecting lines reflects the frequency of co-infection between the two species; thicker lines indicate a higher co-infection frequency. The size of each node represents the overall number of co-infection events involving that species; larger nodes indicate greater involvement in co-infections.

A striking feature of the PPVs viral ecosystem is the high frequency of multi-species co-infections. Of the 212 positive samples, 129 (60.85%) harbored at least two distinct species. The complexity of these mixed infections was substantial: 68 cases (52.71%) involved dual-species infections, and the remaining 61 cases (47.29%) contained three or more species. Underscoring this complexity, seven individual animals were simultaneously co-infected with five or more PPVs species (Figure 2A). Analysis of the co-infection composition revealed that the most prevalent species, namely PPV7, PPV6, and PPV2, were the dominant contributors to complex multi-species infections (Figure 2A). Consistent with this observation, they exhibited the highest pairwise co-infection frequency (Figure 2B). To disentangle this prevalence effect and identify species with a disproportionate tendency to co-infect, we calculated the normalized node strength for each species (see Section "Data processing and statistical analysis"). This analysis yielded a key insight; despite their low individual prevalence, PPV1 and PPV5 exhibited the highest normalized strength values in the dataset (Figure 2B). A permutation test confirmed that the high co-infection propensity of the low-prevalence species PPV1 (*p* < 0.001) and PPV5 (*p* = 0.002) was statistically significant, indicating non-random co-occurrence with other species (Additional file 3).

### Genetic divergence and phylogenetic landscape of circulating PPVs

**Figure 3:**
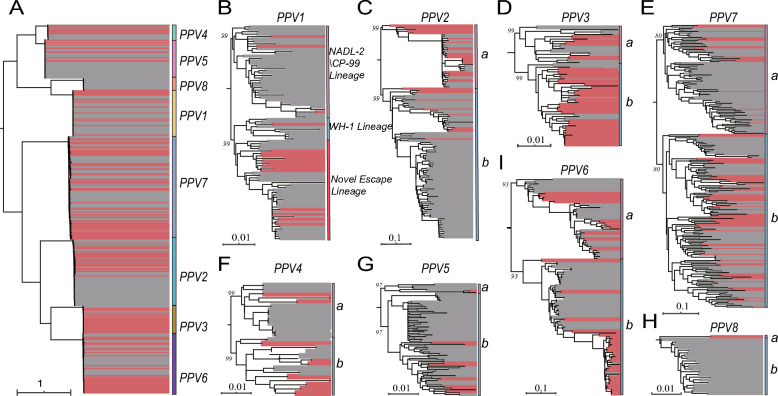
**Global and species-specific phylogenetic trees of PPVs**. Interspecies and intraspecies phylogenetic relationships in PPVs. Each phylogenetic tree was constructed using the maximum likelihood method based on complete coding sequence (CDS) data. All trees were midpoint-rooted and arranged in decreasing order for better visualization. Sequences obtained in this study are highlighted in red. The corresponding scale bars, species, and lineages are labeled adjacent to each tree. A Phylogenetic reconstruction of PPVs species 1 through 8 based on the complete CDS dataset. B–H Individual phylogenetic trees for each of the eight PPV species constructed from their respective CDS datasets. The fully annotated trees and their sequence names are shown in Additional file 4.

Analysis of the complete coding sequences (CDSs) revealed a distinct genetic architecture of the PPVs species. Intra-species nucleotide similarity (88.5–99.9%) was much higher than inter-species similarity (30.1–77.5%), highlighting a clear genetic separation between them (Table 2, Additional file 1). Global phylogeny confirmed these genetic boundaries by clustering all strains into eight well-supported monophyletic groups (Figure 3A). This global perspective underscores the deep evolutionary separations between species, with the single PPV8 strain being particularly noteworthy. The phylogeny of this strain reveals a novel, deeply divergent lineage (Figure 3H).

**Table 2:** **Identity of PPVs at inter-species and intra-species level**

	PPV1	PPV2	PPV3	PPV4	PPV5	PPV6	PPV7	PPV8
PPV1	91.8–99.6	39.9–47.5	43.8–50.3	44.0–52.9	44.3–53.5	34.3–41.7	42.9–49.4	64.4–73.4
PPV2	39.9–47.5	92.5–99.9	58.7–63.9	42.2–51.8	42.1–51.6	44.6–50.5	43.5–50.7	36.5–46.0
PPV3	43.8–50.3	58.7–63.9	95.0–99.7	45.3–54.8	45.5–54.3	46.0–51.4	45.6–50.4	41.6–49.7
PPV4	44.0–52.9	42.2–51.8	45.3–54.8	88.5–99.9	68.0–77.5	42.0–51.6	39.8–45.5	43.3–51.7
PPV5	44.3–53.5	42.1–51.6	45.5–54.3	68.0–77.5	91.4–99.9	41.8–51.6	40.8–46.5	43.7–52.9
PPV6	34.3–41.7	44.6–50.5	46.0–51.4	42.0–51.6	41.8–51.6	92.3–99.9	33.6–40.8	30.1–40.0
PPV7	42.9–49.4	43.5–50.7	45.6–50.4	39.8–45.5	40.8–46.5	33.6–40.8	93.8–99.7	42.4–48.5
PPV8	64.4–73.4	36.5–46.0	41.6–49.7	43.3–51.7	43.7–52.9	30.1–40.0	42.4–48.5	91.0–99.8

Further examination of the phylogenies revealed two primary modes of evolution (Figure 3B–I). The most common mode observed in the non-vaccine-targeted species, such as PPV3, PPV6, and PPV7, was lineage coexistence (Figure 3D, E, I). In these groups, the strains were segregated into at least two distinct co-circulating clades, indicative of stable, ongoing diversification under natural selection. In contrast, the phylogeny of PPV1 revealed a striking pattern of lineage replacement, suggesting strong directional or convergent evolution (Figure 3B). This is underscored by the diverse genetic origins of the available vaccines, as the two commercial strains that we sequenced belong to two separate, deep-rooted lineages. Crucially, the vast majority (13/17) of contemporary field isolates formed a single homogeneous monophyletic clade that was genetically distant from all identified vaccine lineages, indicating the successful sweep of this dominant new group. The stark contrast between the stable coexistence of natural species and convergent replacement of vaccine-targeted PPV1 requires a mechanistic explanation. Therefore, we shifted our analytical focus to high-resolution analysis of the VP2 capsid protein to identify the specific selective pressures driving this divergence.

### An adaptive trade-off shapes the PPV1 capsid evolution

**Figure 4:**
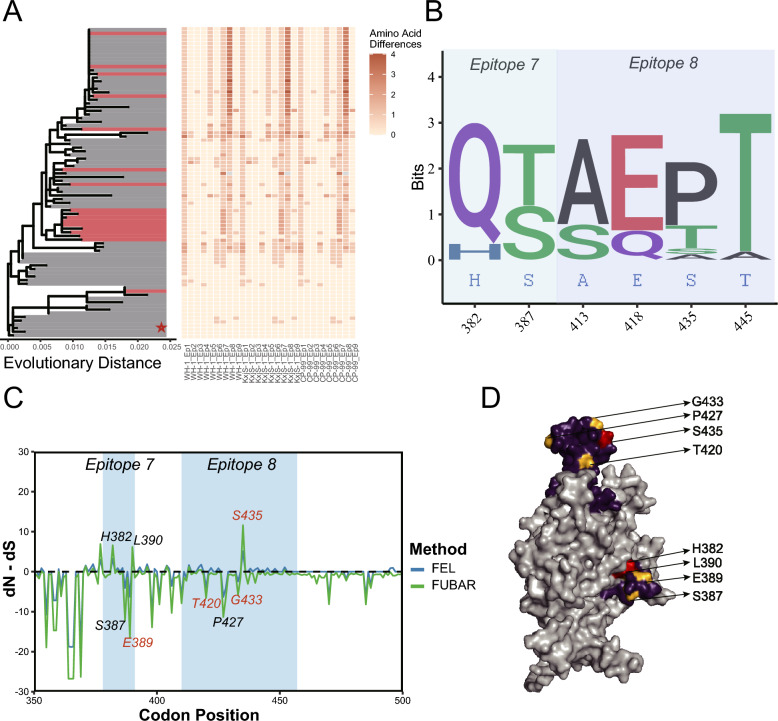
**Phylogenetic, sequence, and structural analysis of the PPV1 VP2 protein**. A High-resolution maximum likelihood phylogenetic tree of PPV1 VP2. The wild type strain clade is highlighted in red, and the reference clade is highlighted in gray. The accompanying heatmap displays amino acid differences relative to the reference sequence, with darker orange indicating greater divergence. Red stars indicate the vaccine strain clade. B Sequence logo of key variable sites within epitopes 7 and 8. Because the epitope sequences of the three reference vaccine strains (WH-1, CP-99, and S-1) were identical, a single consensus sequence is shown in blue. C Site-specific selection pressure analysis (normalized dN-dS) of epitopes 7 and 8 using FEL (blue) and FUBAR (green) methods. The labeled residues in red are supported by both methods, whereas those in black are supported by a single method. D 3D surface model of the PPV1 VP2 protein. Epitopes 7 and 8 are highlighted in purple. Within these epitopes, sites under positive selection are colored red and sites under purifying (negative) selection are colored yellow. The remainder of the protein surface is light gray.

To dissect the mechanisms behind PPV1's unique evolutionary trajectory, we performed a targeted analysis of its *VP2* gene. A high-resolution phylogeny based on this single gene immediately revealed a conflict with the whole-genome tree; while genetically distinct at the genomic level, the commercial vaccine strains clustered together, suggesting a complex evolutionary history (Figure 4A). Formal recombination analysis resolved this incongruence, identifying a clear recombination event in one vaccine strain that possessed a chimeric genome with its *VP2* region likely derived from a different parental lineage (Additional file 2). This finding indicates that circulating field strains have diverged from genetically complex vaccine landscapes, a trend visualized by the amino acid differences in the accompanying heatmap, particularly within epitopes 7 and 8 (Figure 4A).

After clarifying the phylogenetic relationships, we examined the specific selection pressures driving the divergence of the dominant field-strain clade from these vaccine lineages. Our analysis revealed a complex mosaic of evolutionary regimes acting on key sites (Figure 4B, C). Site 435 represents a canonical case of successful antigenic drift in which a near-complete amino acid substitution was driven by unambiguous positive selection. Other sites exhibited more complex dynamics: the substitution at site 382 was associated with only a marginal signal of positive selection, while site 387, despite a clear shift in its dominant amino acid (S → T), was paradoxically identified as being under purifying selection, suggesting an intense conflict between adaptive pressure and functional constraint.

These dynamic sites, under positive or conflicting selection, were juxtaposed with a stable backbone of highly conserved positions. Three sites (residues 389, 420, and 433) were identified under strong, unambiguous purifying selection, likely forming the absolute structural core of the protein. Mapping of these functionally distinct sites onto the three-dimensional (3D) structure illustrates this evolutionary dynamic (Figure 4D). Collectively, this detailed analysis revealed a sophisticated adaptive trade-off: PPV1 achieves antigenic escape not through random mutation, but by sculpting surface-exposed epitopes via positive selection, while strong purifying selection simultaneously preserves the structural integrity of the capsid core.

### Intra-species recombination is frequent and concentrated in the capsid region

Phylogenetic analysis revealed that the dominant PPV species (PPV2, PPV6, and PPV7) were characterized by the co-circulation of multiple distinct lineages (Figure 3). This high intra-species diversity creates a fertile environment for cellular co-infection with genetically different strains of the same type, providing frequent opportunities for genetic recombination. Our analysis confirmed this finding by showing that intra-species recombination occurred exclusively in these prevalent species, with PPV7 exhibiting the highest frequency. In contrast, no inter-species recombination events were detected, suggesting robust evolutionary barriers between species.

**Figure 5:**
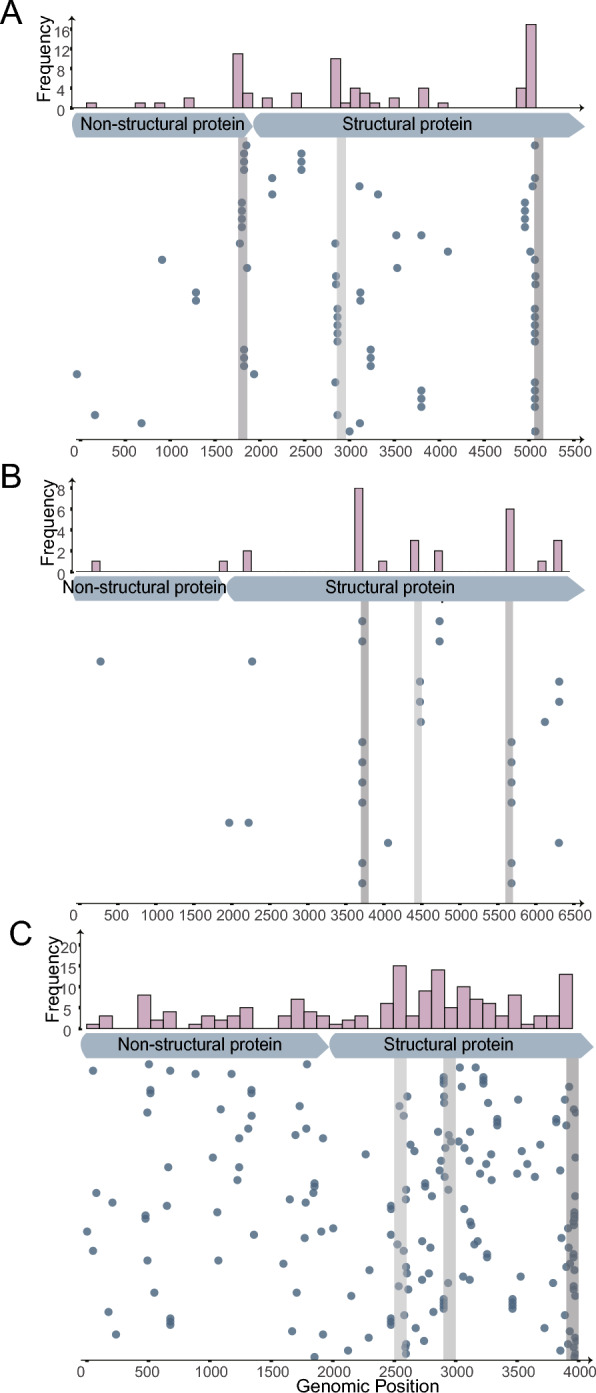
**Analysis of intra-species recombination breakpoints in PPV2, PPV6, and PPV7**. Distribution of intra-species recombination breakpoints. Breakpoints on each horizontal line originated from a recombination event, and the ORFs of the different PPV species are shown below. Furthermore, the frequency of recombination in the genomic regions of each species was calculated, and the hotspot regions are colored purple. A PPV2, B PPV6, and C PPV7.

Breakpoint mapping revealed that the recombination events were not randomly distributed across the genome (Figure 5). For all three species, hotspots were overwhelmingly concentrated within the *ORF2* locus, which encodes the primary antigenic target, the viral capsid. Although some breakpoints were identified in the more conserved nonstructural gene (*NS1*), this striking concentration of recombination events within the capsid-coding region provides compelling evidence that recombination is a key adaptive strategy for these naturally circulating viruses. It is a powerful engine for generating novel antigenic surfaces that evade diverse and constantly shifting immune pressures in the host population.

### Hypervariable hotspots on the viral capsid preferentially target immune epitopes

**Figure 6:**
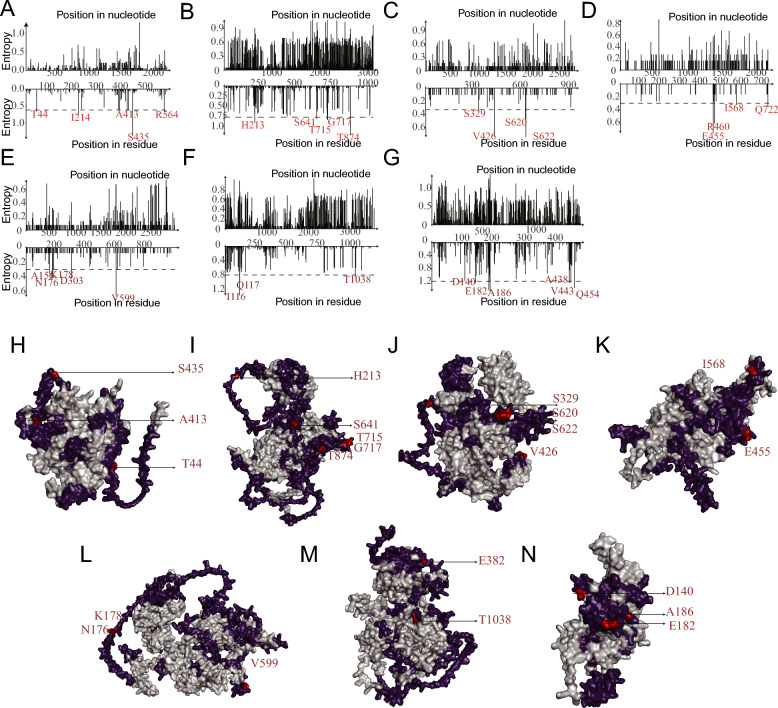
**Shannon entropy analysis of capsid proteins and mapping of hypervariable hotspots**. A–G Variations in entropy and position in the structural protein of seven species of PPV at the nucleotide and residue levels. Hotspots with a high likelihood of mutagenesis and entropy are marked in red. H–N Three-dimensional (3D) structures of seven PPV species. The specific panels correspond to the following viral species and reference structures: (A, H) PPV1 NADL-2 (GenBank: NC001718.1), (B, I) PPV2 Brazil (GenBank: KY586144.1), (C, J) PPV3 Brazil (GenBank: KY586145.1), (D, K) PPV4 (GenBank: GQ387499), (E, L) PPV5 MI216 (GenBank: JX896318.1), (F, M) PPV6 (GenBank: KY094494.1), and (G, N) PPV7-GX23-1998 (GenBank: MN326273.1). Elements colored purple represent potential epitopes, and elements colored red represent hotspots located in the potential epitopes.

To quantify the sequence variability across the viral capsid, we calculated the Shannon entropy for the structural protein-coding region of each major PPV species. This analysis revealed that synonymous mutations were more frequent than non-synonymous mutations, suggesting that strong purifying selection acts on most capsid proteins (Figure 6A–G). The overall diversity varied among species, with PPV7 exhibiting the highest average entropy (0.136) and PPV4 the lowest (0.011), consistent with their phylogenetic patterns (Additional file 5).

To identify the most hypervariable regions, we identified 32 mutational hotspots across seven major species, based on their high Shannon entropy values. Mapping these hotspots onto predicted 3D structures revealed their clear biological significance; 68.75% (22/32) were located directly within the predicted linear B cell epitopes (Figure 6H–N). This pattern was particularly striking in PPV2 and PPV3, where high-entropy sites, such as T874 (PPV2) and T182/N186 (PPV7), were consistently found on the surface of the predicted epitopes. Collectively, this pronounced clustering of the most variable sites within known antigenic regions provides strong evidence that host immune pressure is the primary driver of PPV capsid evolution.

## Discussion

This study delineated the complex molecular epidemiology of PPV in China by characterizing a shifting viral ecosystem at an unprecedented resolution. Our systematic surveillance established an overall PPV detection rate of 24.57% (212/863 samples; 95% CI: 21.7%–27.6%)., a conservative figure that likely reflects the use of serum samples for viremia detection versus tissue-based approaches [19]. The most critical finding is the profound restructuring of this ecosystem; classical PPV1 is no longer the dominant pathogen and has been displaced by a new consortium of species (PPV7, PPV6, PPV3, and PPV2). This ecological transition mirrors trends across Asia and Europe, confirming it as a global phenomenon [18, 19, 54]. The structure of this new landscape is defined by pervasive co-infection (61.14%), which substantially exceeds that reported previously [13, 18, 19, 55]. This highlights not only the enhanced resolution of Next-Generation Sequencing (NGS) but also reveals that the true complexity of viral community interactions has been historically underestimated, providing the essential ecological context for the evolutionary dynamics we subsequently explored.

One of the most thought-provoking findings of this study was that despite their low individual prevalence, PPV1 and PPV5 exhibited the strongest, statistically validated propensity for co-infection. This nonrandom pattern of association suggests complex biological interactions that extend beyond simple stochastic co-occurrence. This may be driven by several mechanisms. In China, PPVs control in commercial breeding herds predominantly relies on inactivated PPV1 vaccines; licensed seed strains reported include WH-1, L, SC1, CG-05, NJ, BJ-2 and YBF01, alongside inactivated VP2-based vectored/subunit products [56]. Vaccination is generally administered intramuscularly to breeding females, with primary immunization completed before first mating in replacement gilts and boosters in sows according to farm/product protocols; protective immunity is expected after completion of the primary course (commonly a two-dose schedule with a ~3-week interval). Because these programs mainly reduce SMEDI rather than fully blocking subclinical infection or shedding, PPV1 may continue to circulate and co-occur with other species, providing ecological context for the frequent multi-species co-infections observed here [56]. First, a “pioneer infection” by a highly prevalent species (e.g., PPV7) might transiently suppress the host's immune response [5], creating a window of opportunity for “follower infections” by PPV1 and PPV5. Second, the evolution of PPV1 under vaccine pressure may have incurred a fitness cost [57], making it more dependent on co-infection with a more fit naturally circulating strain to leverage its replication machinery or immune evasion proteins, thereby forming a synergistic interaction [58]. These hypotheses provide a clear direction for future research on interviral interactions, specifically the need to move from genomic data to functional validation.

The extensive genetic diversity observed in our study was fueled by distinct mechanisms that exemplified the two primary evolutionary modes operating across this ecosystem. For the dominant, naturally-circulating species, evolution is driven by a potent “engine of diversity”. This process is fundamentally underpinned by the high mutation rate inherent to ssDNA viruses such as PPV, which lack a proofreading polymerase and thus provide abundant raw material for evolution [59, 60]. Our analysis revealed that this potential is realized through frequent intra-species recombination, which is concentrated in antigenic ORF2. By shuffling genetic material within this primary antigenic region, recombination serves as a key mechanism for generating novel viral surfaces that navigate the diverse natural immune pressures of the host population [61]. The central role of host immunity as this landscape's primary selective force was further underscored by our entropy analysis, which revealed that the majority of mutational hotspots across all species were concentrated within predicted B-cell epitopes. This pattern is consistent with classic models of immune-driven antigenic drift [62].

In contrast, the evolution of vaccine-targeted PPV1 exemplified a powerful “selective filter.” This long-term, PPV1-focused vaccination background may also contribute to the reduced population prevalence of PPV1 observed in our survey while preferentially selecting for antigenically drifted PPV1 variants that persist in a multi-species ecosystem [28, 56, 63]. Here, the evolutionary solution is not broad diversification but a sophisticated adaptive trade-off within its VP2 capsid, where intense positive selection on specific, surface-exposed epitopes is balanced by strong purifying selection on the structural core. This precise molecular strategy offers a compelling explanation for the re-emergence of PPV1-associated clinical diseases in vaccinated herds. For example, recent studies in Europe have reported that novel 27a-like PPV1 strains were responsible for reproductive failure in vaccinated swine, demonstrating that vaccine-induced immunity may not fully protect against these divergent field strains [28]. Our findings strongly support the conclusion that this phenomenon is driven by antigenic drift, a classic evolutionary pathway for viruses under sustained vaccine pressure [57].

This study has several limitations that warrant acknowledgment. First, our analysis based on serum samples provides a snapshot of systemic viremia and may have underestimated the prevalence of infections localized in specific tissues. Second, ongoing surveillance remains crucial to ascertain the clinical relevance of rare species, such as the highly divergent PPV8 lineage we identified, and to monitor the emergence of entirely novel ones. Furthermore, fundamental biological characteristics such as pathogenicity and tissue tropism of many of the now-dominant non-PPV1 species remain poorly understood, which underscores the importance of such research. Finally, individual-level vaccination dates and sampling times relative to vaccination were unavailable, precluding direct evaluation of time-to-immunity or vaccine–co-infection causal links. Most importantly, although our genomic data provide compelling evidence for the evolutionary mechanisms driving antigenic changes, experimental challenge models and serological neutralization assays are urgently required to quantify the precise impact of these mutations on in vivo vaccine efficacy.

## Conclusion

In conclusion, by deconstructing a complex viral ecosystem using a comparative eco-evolutionary framework, this study moved beyond simple prevalence reporting to delineate the multifaceted dynamics of PPV. By linking epidemiological patterns to specific molecular mechanisms, such as adaptive trade-offs under vaccine pressure and the role of recombination in generating natural diversity, our study provides a new paradigm for understanding how viruses adapt and thrive in managed host populations. These findings underscore the urgent need to reevaluate current control strategies and lay an essential scientific foundation for the rational design of next-generation surveillance programs and broadly protective multivalent vaccines.

## Supplementary Information

**Additional file 1**. **Heatmap showing the identity of PPVs at the inter-species and intra-species levels.**

**Additional file 2**. **Evidence for a recombination event in a commercial PPV1 vaccine strain.**

**Additional file 3**. **Co-infection network analysis and permutation test results for each PPV species.**

**Additional file** **4**. **GenBank ID of reference sequences used in this study.**

**Additional file** **5**. **Entropy at each position of the eight PPV species and results of antigenic epitope prediction.**

## Data Availability

The datasets used and/or analysed during the current study are available from the corresponding author on reasonable request.
