# Supplementary figures and images for: Eco-evolutionary dynamics of a shifting porcine parvoviruses (PPV1–PPV8) ecosystem reveal dichotomous selection pressures
Source: Vet Res. 2026 Apr 18;57:91. doi: 10.1186/s13567-026-01756-4 (PMC13214466; doi:10.1186/s13567-026-01756-4)

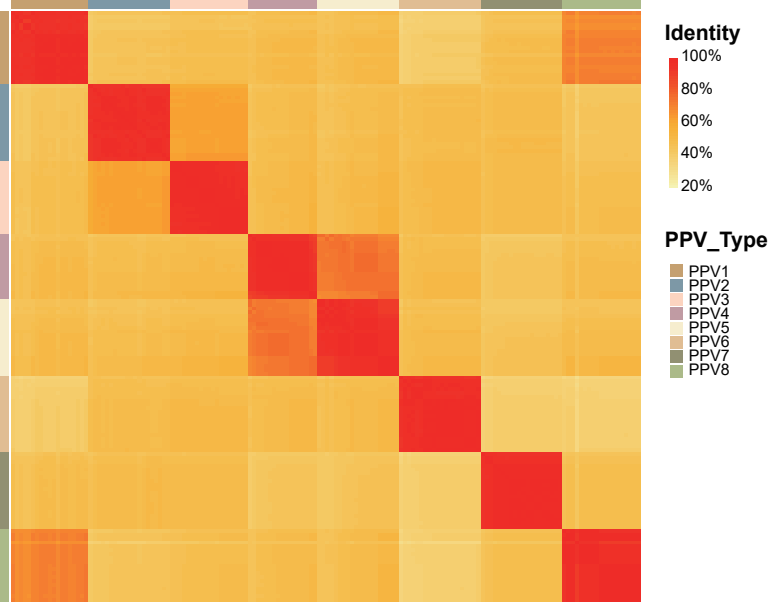

Supplement: Supplementary file 1 — Additional file 1. Heatmap showing the identity of PPVs at the inter-species and intra-species levels. [file 13567_2026_1756_MOESM1_ESM.pdf]

Sequence Identity

NC\_001718.1\_NADL-2

PV296190.1\_PPV1-S2VM-ABT-2025

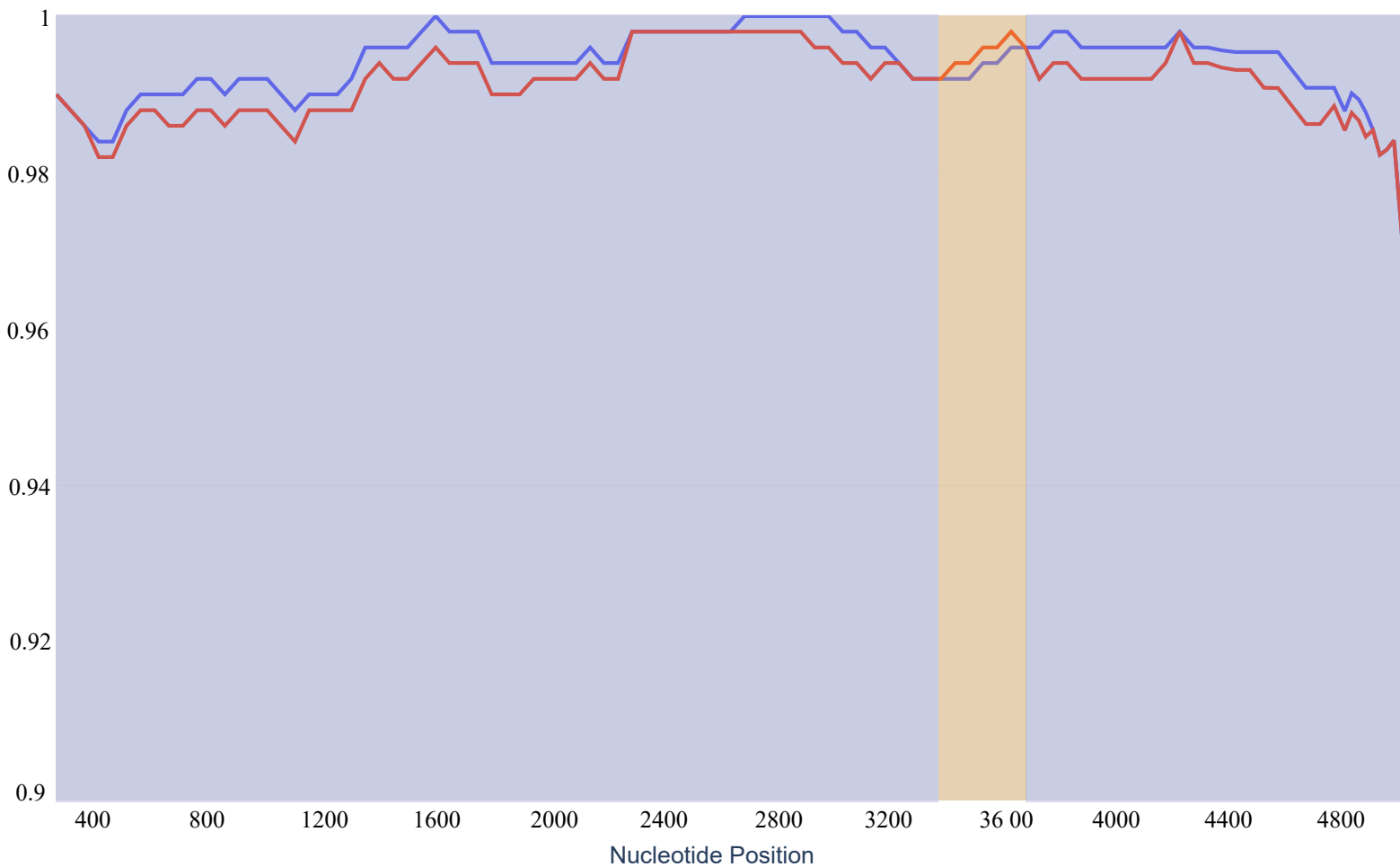

NSP1

VP2

VP1

Supplement: Supplementary file 2 — Additional file 2. Evidence for a recombination event in a commercial PPV1 vaccine strain. [file 13567_2026_1756_MOESM2_ESM.pdf]
